# Supplementary figures and images for: The Association of Metformin, Other Antidiabetic Medications, and Statins with the Prognosis of Hepatocellular Carcinoma in Patients with Type 2 Diabetes: A Retrospective Cohort Study
Source: Biomedicines. 2024 Jul 24;12(8):1654. doi: 10.3390/biomedicines12081654 (PMC11351525; doi:10.3390/biomedicines12081654)

A. Insulin

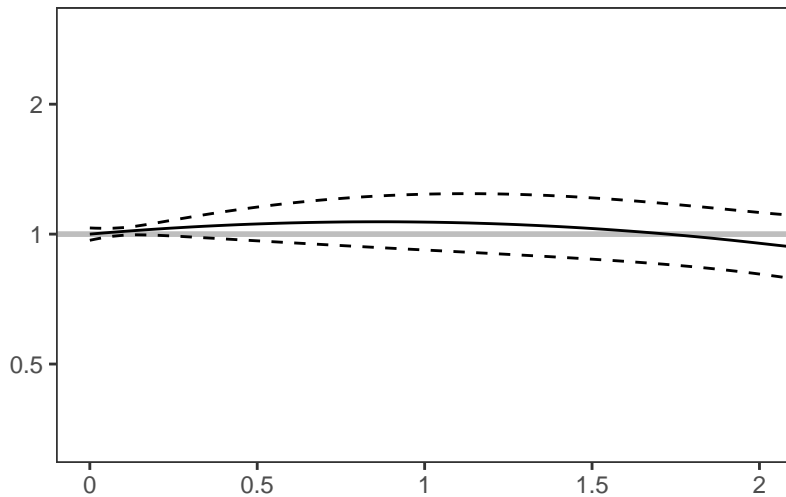

B. Metformin

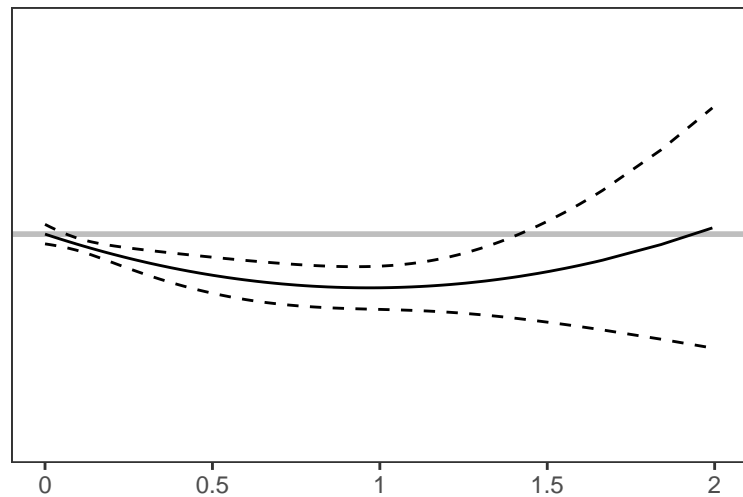

C. Other

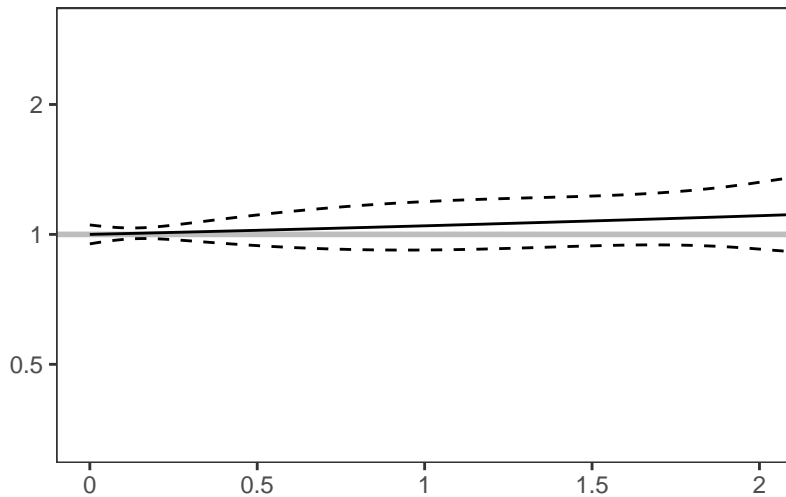

D. Statin

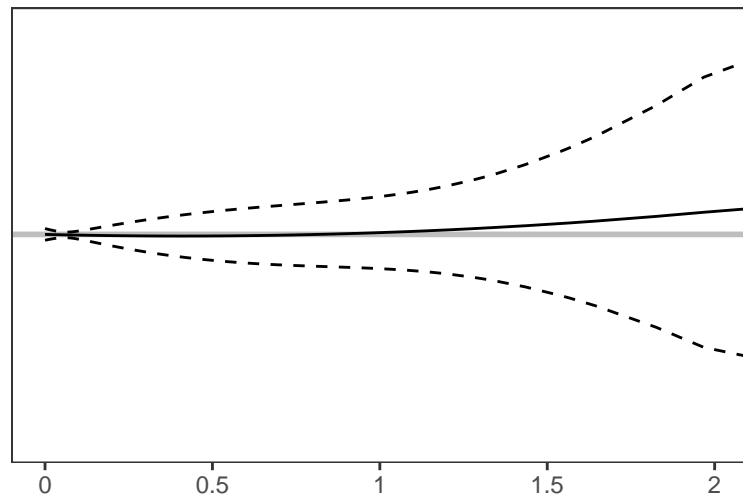

Average DDD use during 3 years period

Supplement: Supplementary file 1 [file biomedicines-12-01654-s001.zip › Supplementary files 240724/Figure S1.pdf]

A. Insulin

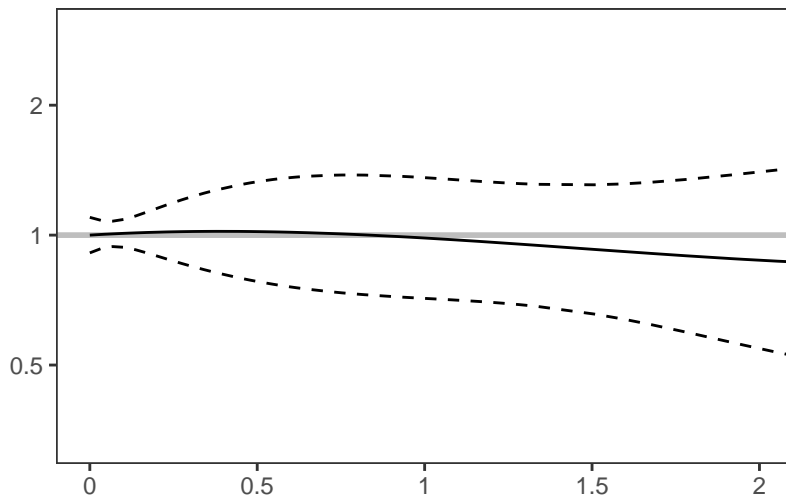

B. Metformin

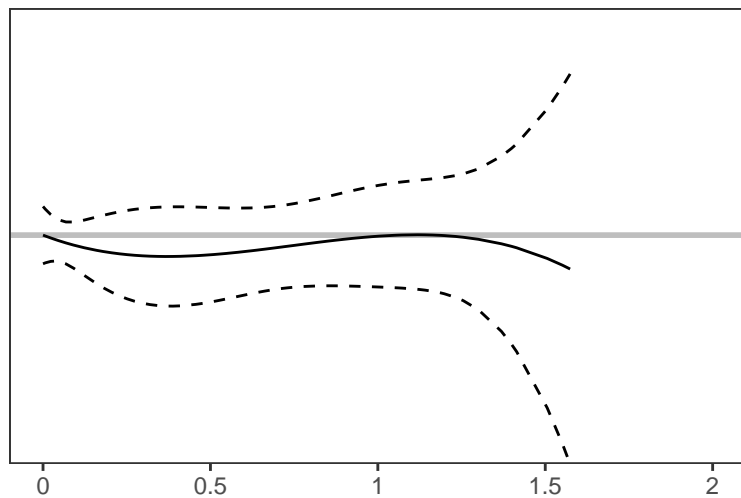

C. Other

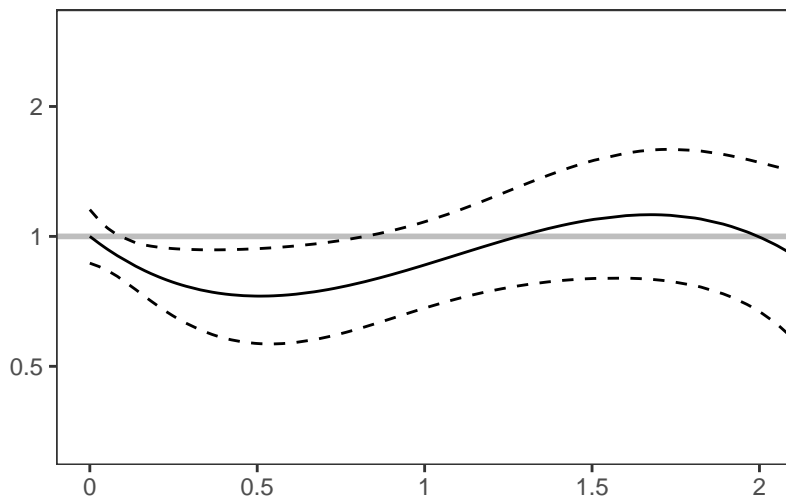

D. Statin

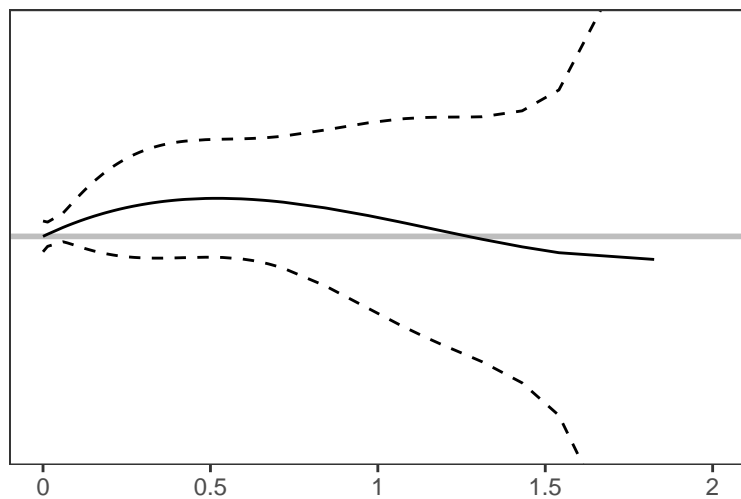

Average DDD use during 3 years period

Supplement: Supplementary file 1 [file biomedicines-12-01654-s001.zip › Supplementary files 240724/Figure S2.pdf]
